# Nitric Oxide and Superoxide Anion Balance in Rats Exposed to Chronic and Long Term Intermittent Hypoxia

**DOI:** 10.1155/2014/610474

**Published:** 2014-02-26

**Authors:** Patricia Siques, Ángel Luis López de Pablo, Julio Brito, Silvia M. Arribas, Karen Flores, Karem Arriaza, Nelson Naveas, M. Carmen González, Alexander Hoorntje, Fabiola León-Velarde, M. Rosario López

**Affiliations:** ^1^Instituto de Estudios de la Salud, Universidad Arturo Prat, Avenida Arturo Prat 2120, 11100939 Iquique, Chile; ^2^Departamento de Fisiología, Facultad de Medicina, Universidad Autónoma de Madrid, 28029 Madrid, Spain; ^3^Instituto de Investigación Hospital Universitario Gregorio Marañón, 28007 Madrid, Spain; ^4^Departamento de Ciencias Biológicas y Fisiológicas, Facultad de Ciencias y Filosofía/IIA, Universidad Peruana Cayetano Heredia, Lima 31, Lima, Peru; ^5^Departamento de Medicina Preventiva, Salud Pública y Microbiología, Facultad de Medicina, Universidad Autónoma de Madrid, 28029 Madrid, Spain

## Abstract

Work at high altitude in shifts exposes humans to a new form of chronic intermittent hypoxia, with still unknown health consequences. We have established a rat model resembling this situation, which develops a milder form of right ventricular hypertrophy and pulmonary artery remodelling compared to continuous chronic exposure. We aimed to compare the alterations in pulmonary artery nitric oxide (NO) availability induced by these forms of hypoxia and the mechanisms implicated. Rats were exposed for 46 days to normoxia or hypobaric hypoxia, either continuous (CH) or intermittent (2 day shifts, CIH2x2), and assessed: NO and superoxide anion availability (fluorescent indicators and confocal microscopy); expression of phosphorylated endothelial NO synthase (eNOS), NADPH-oxidase (p22phox), and 3-nitrotyrosine (western blotting); and NADPH-oxidase location (immunohistochemistry). Compared to normoxia, (1) NO availability was reduced and superoxide anion was increased in both hypoxic groups, with a larger effect in CH, (2) eNOS expression was only reduced in CH, (3) NADPH-oxidase was similarly increased in both hypoxic groups, and (4) 3-nitrotyrosine was increased to a larger extent in CH. In conclusion, intermittent hypoxia reduces NO availability through superoxide anion destruction, without reducing its synthesis, while continuous hypoxia affects both, producing larger nitrosative damage which could be related to the more severe cardiovascular alterations.

## 1. Introduction

Exposure to hypoxia, in either chronic or intermittent conditions, is associated with cardiovascular alterations. Hypoxia-related diseases are well characterized in people living at high altitude (chronic hypoxia, CH) [1] and in obstructive sleep apnea (OSA), where hypoxic conditions are maintained intermittently for brief periods [2]. Another mode of intermittent hypoxia has arisen as a result of the recent settlements of mines and other activities at high altitude (chronic intermittent hypoxia, CIH), where subjects repeatedly ascend from sea level to 3800–4200 m and work in shifts, being exposed to hypoxia for longer periods than in OSA conditions [3]. The large number of workers under these circumstances, together with the known deleterious cardiovascular effects reported in OSA patients [4], makes it of utmost importance to study the mechanisms implicated in CIH alterations. Since this condition is a relatively recent phenomenon, studies are still limited and animal models are valuable. We have used an experimental model of CIH, where rats are exposed to hypobaric hypoxia in shifts resembling the human situation [5]. We and others have found that under these experimental conditions the rats develop pulmonary hypertension, pulmonary vascular remodeling, and right ventricular hypertrophy [6, 7], which are milder compared to rats chronically exposed to hypoxia [8].

Hypoxia-induced pulmonary hypertension and right ventricular hypertrophy are related to pulmonary artery vasoconstriction and vascular remodeling [9, 10], endothelial nitric oxide (NO) being an important modulator of these responses [11, 12]. Human and animal studies demonstrate that OSA is associated with reduced NO availability, due to a decreased production or destruction by an excess of reactive oxygen species (ROS) [2] and pulmonary artery vasoconstriction has been suggested to result from imbalance between endothelial vasodilator factors and ROS [13]. NO-ROS misbalance can also contribute to remodeling process in the pulmonary vasculature, through modification of cell migration, proliferation, dedifferentiation, and apoptosis [14]. We have previously demonstrated that rats exposed to long term CIH exhibit pulmonary artery remodeling with characteristic features which differ from those found under chronic exposure conditions [15, 16]. While there are several studies in OSA conditions, to the best of our knowledge, there are no data regarding the effect of hypoxia maintained intermittently during days on NO/ROS balance in the pulmonary vasculature. Therefore, we aimed to analyze the possible alterations under this condition and to compare them with those induced by continuous hypoxic exposure. We have used a rat model mimicking the conditions of subjects working in shifts or permanently living at high altitude.

## 2. Materials and Methods

### 2.1. Animal Model

Three-month-old male Wistar rats were used. The rats were exposed to 22 ± 2°C, 12 h light/dark cycle and were maintained in separate cages, with food made available to them (20 g pellets/day per rat) and water *ad libitum*. Standard veterinary care was used during all of the experiments following institutional protocols for the study of animals and the procedures used were approved by the Institutional Research Ethics Committee of Universidad Arturo Prat (Chile). The rats were randomly assigned to one of the following groups:

 normoxia control group (NX; *n* = 10);
 chronic intermittent hypoxia, 2 days under hypobaric hypoxia and 2 days under normobaric normoxia for 46 days (CIH2x2; *n* = 10);
 chronic hypoxia, continuous hypobaric hypoxia for 46 days (CH; *n* = 10).

Hypobaric hypoxia was simulated at Universidad Arturo Prat facilities in a hypobaric chamber at 428 Torr, which is equivalent to an altitude of 4600 m (PO_2_ = 89 mmHg, PCO_2_ = 0.15 mmHg, temperature 22 ± 2°C, and humidity 35 ± 5%). Control (sea level) conditions were PO_2_ = 159 mmHg, PCO_2_ = 0.29 mmHg, temperature 22 ± 2°C, and humidity 35 ± 5%. The animal model for chronic intermittent hypoxia exposure (CIH2x2) has been described previously [5, 8, 17]. This model involves 2 days of hypoxia and 2 days of normoxia over a period of 46 days. We maintained the rats for 46 days, since previous data indicate that the hematological and cardiovascular effects in the rat are maximal between 30 and 45 days of exposure to hypoxia [5]. The 2x2 regimen was chosen in order to mimic long term exposure to intermittent hypoxic conditions as experienced by human subjects working in shifts at high altitude, which is in the order of days (usually 7–14) [18], and including at least one full circadian cycle. The control group was placed in the same room at sea level during the 46-day period.

All the rats were weighted at day 1 and at day 46 using an Acculab V-1200 electronic balance. At day 46 a blood sample was taken from the tail and hematocrit was measured using a microcentrifuge (Eppendorf AG, Hamburg, Germany). Thereafter, the animals were euthanized with an overdose of anesthesia (Ketamine, 8 mg, i.p.) and the heart and the lungs were removed in a block for further dissection.

The heart was cut down and the right ventricle was detached from the heart, leaving *in situ* the septum portion together with the left ventricle. Both ventricles were weighed in an analytic balance (Acculab V-1200, Illinois, USA) and the ratio between the right ventricle/left ventricle plus the septum weight was used to measure the grade of right ventricular hypertrophy. We did not use total heart weight or right ventricular weight/body weight since we have previously reported that exposure to hypoxia produces alterations in body weight gain [5]. The removed lung was placed in a Petri dish and pulmonary artery branches (4th order) were dissected and stored for further experiments in saline solution (0.9% NaCl).

### 2.2. Confocal Microscopy

#### 2.2.1. Determination of NO Availability

Basal NO availability was determined by the fluorescent NO indicator 4,5-diaminofluorescein diacetate (DAF-2 DA, Sigma) as described previously [19]. Briefly, 3 mm length pulmonary artery segments were stabilized in physiological salt solution (PSS; 115 mmol/L NaCl, 4.6 mmol/L KCl, 25 mmol/L NaHCO_3_, 1.2 mmol/L KH_2_PO_4_, 1.2 mmol/L, MgSO_4_, and 2.5 mmol/L CaCl_2_), for 30 min at 37°C, and oxygenated with carbogen (95% O_2_ and 5% CO_2_). Thereafter, they were stained with oxygenated DAF-2 DA solution (10 *μ*mol/L) for 30 min in the darkness at 37°C, in a shaking water bath. This experimental procedure in saturated oxygen ensures that NO, rather than O_2_, is the limiting factor in the reaction and the fluorescence is directly proportional to NO [19]. Negative controls for DAF-2 DA were incubated in 0.1 mmol/L L-NAME throughout the experimental period. The segments were then washed 3 times for 1 min each in PSS and fixed in 4% (w/v) paraformaldehyde. The segments were cut in rings with a blade and mounted on a slide equipped with a small well made of spacers, filled with mounting medium (Citifluor, Aname, Spain), and covered with a cover glass. The arterial rings were visualized with a Leica TCS SP2 confocal system (Leica Microsystems, Wetzlar, Germany) at Universidad Autónoma de Madrid of Spain facilities using the 488 nm/515 nm line. 1 *μ*m thick serial images (25 *μ*m in total) were captured with a 63x objective at zoom 2 in 3 randomly chosen areas of the ring, at identical conditions of brightness, contrast, and laser power for all of the experimental groups. MetaMorph image analysis software (Universal Imaging Co., UK) was used for quantification of fluorescence intensity. Briefly, the serial images were first reconstructed in a confocal projection and fluorescence intensity was quantified in several regions of the smooth muscle cells, where the dye is trapped, avoiding the elastic lamella which is also fluorescent in the same wavelength.

#### 2.2.2. Determination of O_**2**_^●−^ Availability

Dihydroethidium (DHE, Sigma) was used to determine basal O_2_^∙−^, as described [19]. Briefly, 3 mm long pulmonary arteries were stabilized in PSS (30 min at 37°C). Thereafter, they were incubated with 3 *μ*mol/L DHE, washed 3 times for 1 min each in PSS, and fixed in 4% (w/v) paraformaldehyde. Negative controls for DHE were incubated in 15 units/mL superoxide dismutase (SOD) throughout the incubation period. The segments were then washed 3 times for 1 min each in PSS, fixed in 4% (w/v) paraformaldehyde, cut in rings, and mounted as described above. 1 *μ*m thick serial images (25 *μ*m in total) were captured with a 63x objective at zoom 2 in 3 randomly chosen areas of the ring, at identical conditions of brightness, contrast, and laser power for all of the experimental groups with the 488 nm/590–620 nm line of the microscope. MetaMorph image analysis software (Universal Imaging Co., UK) was used for quantification of fluorescence intensity, which was located in the nuclei. The serial images were first reconstructed in a confocal projection and fluorescence intensity was quantified in several regions along the ring.

#### 2.2.3. NADPH-Oxidase Detection by Immunohistochemistry

To detect the presence of NADPH oxidase in the adventitial layer, pulmonary arteries were first incubated with the primary antibody of the p22phox subunit of the enzyme (rabbit polyclonal, Santa Cruz Biotechnology, USA) (60 min, 1 : 200 in saline solution) at room temperature (RT) and then washed with saline solution (30 min, RT). Thereafter, the segments were incubated with the secondary antibody-Alexa Fluor 647 goat anti-rabbit IgG (H + L) (Invitrogen, Madrid, Spain) (60 min, 1 : 200, RT in the darkness), followed by washing for 30 min, RT. Finally, they were incubated with the nuclear dye 4′,6-diamidino-2-phenylindole (DAPI; 1 : 500 from a 5 mg/mL stock; 30 min, RT in the darkness) and washed twice (30 min, RT). The pulmonary arteries were longitudinally sectioned and mounted with the adventitial side facing up, as described above. The arteries were visualized with a Leica TCS confocal system using the 405 nm excitation/410–475 nm emission wavelength (DAPI) to locate the cells and the 633 nm excitation/640–650 nm emission wavelength (secondary antibody-Alexa 647) to detect the protein. 1 *μ*m thick serial images of the adventitial layer (12 *μ*m in total) were captured at both wavelengths with a 40x objective at zoom 4 from 3 different regions. MetaMorph software was used to count the total number of cells (DAPI positive) and those stained with p22phox. Cell number (DAPI or p22phox positive) was counted always in the same volume which was calculated from the layer thickness (12 *μ*m) and the image area at ×40 zoom 4. We calculated the total number of p22phox positive cells and the relative number of positive cells (positive cells/total cells) in the mentioned volume.

### 2.3. Western Blot

Western blot was performed according to the standard methods. Briefly, total pulmonary artery tissues were frozen in liquid nitrogen and then homogenized into homogenization buffer containing 50 mM Tris-HCl, 150 mM NaCl, 100 mM NaF, 1% Triton X-100, 1 mM dithiothreitol, 0.1 mM phenyl methylsulfonyl fluoride, 1 mM leupeptin, 0.02 M Hepes, 10^−3^ M EDTA, 10^−3^ M EGTA, and 20% glycerol, followed by centrifugation to 10000 ×g by 10 min. Supernatant was removed and protein quantification was carried out by Bradford assay. Equal amount of protein (25 *μ*g) was resolved on 7.5–12% SDS-PAGE and proteins were transferred to a PVDF membrane. The nonspecific binding sites on the membrane were blocked using 5% nonfat dry milk in TBS-T buffer (10 mM Tris-HCl, 150 mM NaCl, 0.05% tween 20, and pH 7.4) by 1 hour. Membranes were incubated with primary antibody rabbit polyclonal to PNK (3-nitrotyrosine) and p22phox and goat polyclonal pNOS-3 (phosphorylated form of e-NOS; Santa Cruz Biotechnology, USA) by overnight and washed three times for 15,10,5 min with TBS-T followed by incubation with horseradish peroxidase-conjugated secondary antibody (1 : 2000, Santa Cruz Biotechnology, USA) for 1 hour and washed 3 times with TBS-T and once with TBS. Blots were visualized using a West Pico Chemiluminescence System (Pierce, USA) and then analyzed using Image J. Expression levels of p22phox, PNK, and pNOS3 were normalized to *β*-actin expression.

### 2.4. Statistical Analysis

The experimental data were entered into a database and were analyzed using SPSS 17.0 statistical package (SPSS, Inc., Chicago, Ill, USA). Mean, standard deviation, and standard error were calculated for each parameter. Normality was established using the Kolmogorov-Smirnov test. Statistical analysis of the differences across all testing conditions was established using analysis of variance (ANOVA) of one factor and less significant difference post hoc tests. All of the variables were normally distributed. Statistical significance was established at a *P* value < 0.05.

## 3. Results

### 3.1. Body Weight Gain, Hematocrit, and Right Ventricular Hypertrophy

Body weight at day 1 was not statistically different between experimental groups (NX: 247.8 ± 16.6 gr, CIH2x2: 246.9 ± 11.2 gr, and CH: 251.5 ± 17.2 gr). While NX rats gained weight over the 46-day period (final body weight NX = 330 ± 13.5 g), there was a gradual weight loss in both hypoxic groups (final body weight CIH2x2 = 206 ± 8.03 g; CH = 169 ± 3.6 g), being significantly smaller compared to NX (*P* < 0.001). Hematocrit at the end of experimental period was significantly higher in CH (66 ± 1.1%) and in CIH2x2 (58 ± 1.8%), compared to NX rats (51 ± 1.0%) (*P* < 0.01). CIH2x2 hematocrit was significantly smaller compared to CH (*P* < 0.01). Right ventricular weight/total heart weight was smaller in NX (NX = 0.23 ± 0.02; *P* < 0.01) compared to CIH2x2 rats (0.34 ± 0.02) and CH group (0.40 ± 0.02), which was also significantly larger compared to intermittent exposure group (*P* < 0.05).

### 3.2. NO Availability

**Figure 1:**
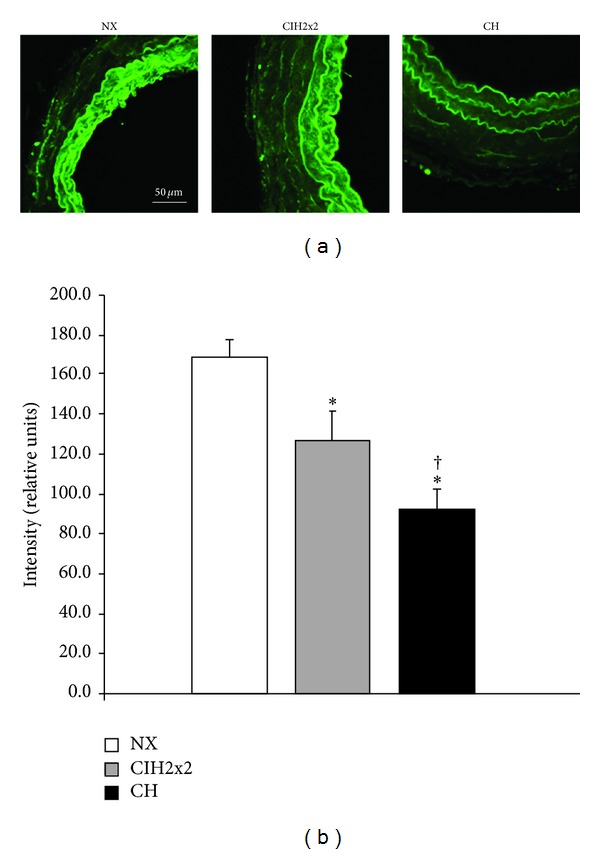
DAF-2 DA intensity levels in pulmonary arteries from rats exposed to normoxia (NX, *n* = 10), intermittent hypoxia (CIH2x2, *n* = 10), or chronic hypoxia (CH, *n* = 10). (a) Representative examples of projections obtained from confocal microscopy images (×40 zoom 2). (b) Quantitative analysis; *n* represents the number of animals; *P < 0.05 compared to NX; ^†^ *P* < 0.05 compared to CIH2x2.

The fluorescence emitted by DAF-2 DA was located in the cytoplasm of smooth muscle cells. Pulmonary arteries from NX group exhibited a significantly higher DAF-2 DA emitted fluorescence compared to both hypoxic groups, suggesting a larger basal NO availability [19]. Fluorescence was significantly lower in CH rats compared to CIH2x2 (Figure 1).

### 3.3. O_**2**_^●−^ Availability

**Figure 2:**
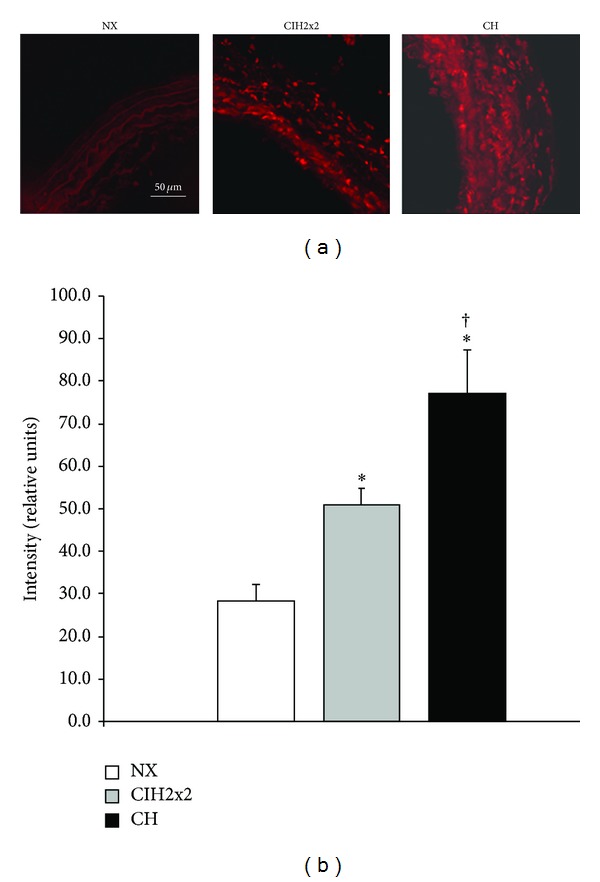
DHE intensity levels in pulmonary arteries from rats exposed to normoxia (NX, *n* = 10), intermittent hypoxia (CIH2x2, *n* = 10), or chronic hypoxia (CH, *n* = 10). (a) Representative examples of projections obtained from confocal microscopy images (×40 zoom 2). (b) Quantitative analysis; *n* represents the number of animals; *P < 0.05 compared to NX; ^†^ *P* < 0.05 compared to CIH2x2.

DHE fluorescence was located in the cell nuclei. NX rats showed a smaller level of fluorescence intensity compared to both hypoxic groups, being higher in CH compared to CIH2x2 (Figure 2).

### 3.4. Phosphorylated eNOS, p22phox, and 3-Nitrotyrosine Expression

**Figure 3:**
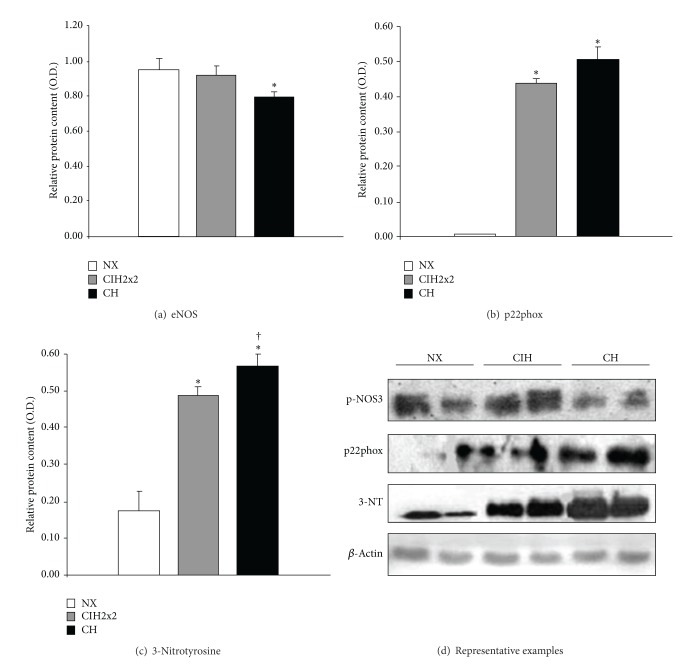
Western blot analysis of: (a) phosphorylated eNOS, (b) p22phox, and (c) 3-nitrotyrosine from pulmonary arteries of rats exposed to normoxia (NX, *n* = 9), intermittent hypoxia (CIH2x2, *n* = 10), or chronic hypoxia (CH, *n* = 10). (d) Representative examples. *n* represents the number of animals; *P < 0.05 compared to NX; ^†^ *P* < 0.05 compared to CIH2x2.

Expression of the phosphorylated form of eNOS was significantly reduced in CH compared to NX. The expression levels in CIH2x2 were not statistically different from NX (Figure 3(a)).

p22phox expression was scarcely detectable in pulmonary arteries from NX but was largely increased in both hypoxic rats, without differences between CH and CIH2x2 groups (Figure 3(b)).

3-Nitrotyrosine expression, a marker of nitrosative damage, was significantly elevated in pulmonary arteries from hypoxic rats compared to NX, being significantly larger in CH compared to CIH2x2 (Figure 3(c)).

### 3.5. p22phox Location in the Adventitia

The total number of adventitial cells, quantified by the nuclear dye DAPI in a fixed volume, was significantly higher in pulmonary arteries from both hypoxic rats (CIH2x2 = 144.7 ± 13.2; CH = 157.1 ± 9.7) compared to control (NX = 74.9 ± 2.6, *P* < 0.001).

**Figure 4:**
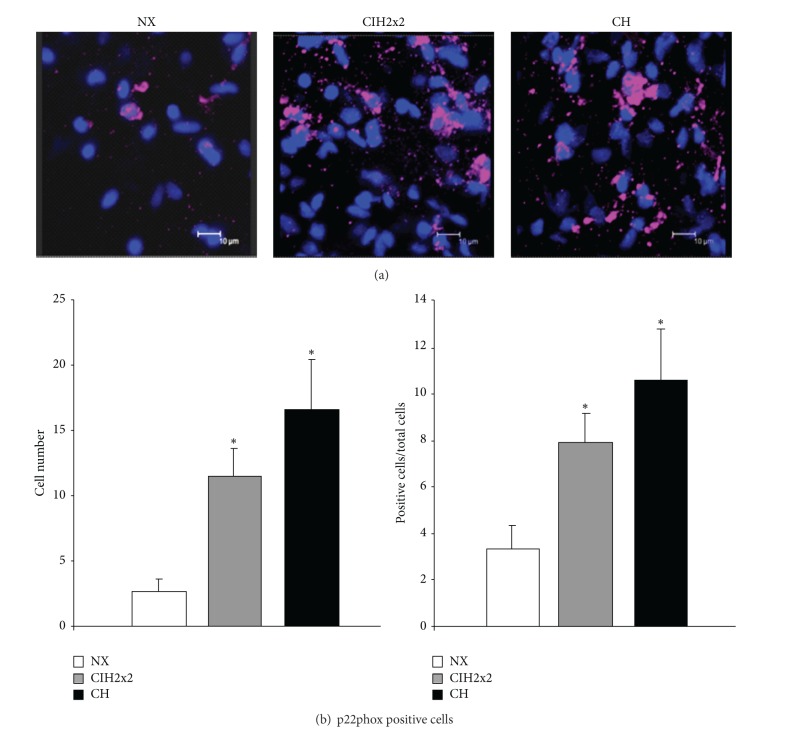
Immunohistochemical detection of p22phox positive cells in the adventitia of pulmonary arteries from rats exposed to normoxia (NX, *n* = 10), intermittent hypoxia (CIH2x2, *n* = 10), or chronic hypoxia (CH, *n* = 10). (a) Representative examples of projections obtained from confocal microscopy images (×40 zoom 4). (b) Quantitative analysis; *n* represents the number of animals; *P < 0.05 compared to NX.

P22phox staining was observed in the three experimental groups and was located around some of the adventitial cells. Pulmonary arteries from hypoxic rats exhibited a larger level of staining compared to NX (Figure 4(a)). Both total and relative numbers of p22phox positive cells were significantly larger in the adventitial layer of hypoxic rats compared to NX, with no statistical difference between CIH2x2 and CH rats (Figure 4(b)).

## 4. Discussion

**Figure 5:**
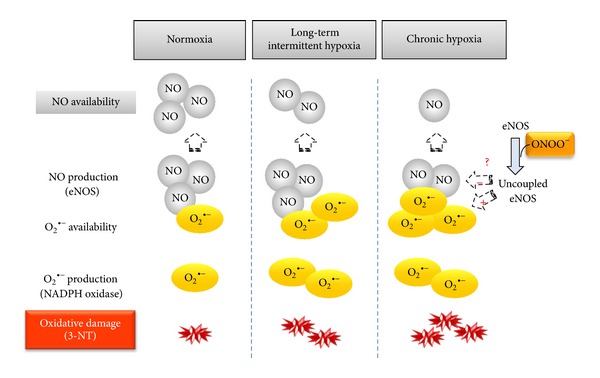
Schematic diagram showing the main results and the proposed mechanism implicated in the NO/O_2_^∙−^ misbalance induced by chronic and intermittent hypoxia.

The main findings of the current study are that exposure to chronic intermittent hypoxia reduces NO availability in the pulmonary vasculature. This decrease is likely due to NO destruction by O_2_^∙−^, generated by NADPH-oxidase, while eNOS is not altered. On the other hand, in continuous exposure to hypoxia, NO availability is further reduced, through the combination of diminished NO synthesis and increased destruction. In consequence, chronic hypoxia produces larger nitrosative damage compared to intermittent exposure, which likely contributes to the higher impact on pulmonary artery remodeling and right ventricular hypertrophy (Figure 5 summarizes these results).

The present data confirmed that hypoxia induced weight loss, right ventricular hypertrophy, and hematocrit increase, as previously described by us [8] and others [6, 7]. We have also described remodeling of the pulmonary vasculature [15, 16], these alterations being less severe under intermittent exposure. We aimed to assess if the above mentioned cardiovascular alterations are linked to NO/ROS misbalance. To the best of our knowledge, there is virtually no information in long term intermittent hypoxia conditions, the majority of evidence coming from OSA studies [20], where low oxygen levels are maintained for very brief periods.

We focused on NO, a key factor for pulmonary artery resistance [12], since diminished NO availability likely affects both pulmonary artery structure and function. Furthermore, studies on rodents have revealed that intermittent exposure to hypoxia is associated with NO reduction in the systemic and cerebral vasculature [21, 22]. Decreased NO availability in the pulmonary vasculature can be the result of a reduced production by eNOS and/or an increased destruction by ROS, particularly O_2_^∙−^, which has been implicated in hypoxic pulmonary vasoconstriction [13, 23–25]. To determine NO availability, we used DAF-2 DA, a fluorescent indicator directly proportional to the amount of NO [26, 27]. Using confocal microscopy and image analysis software, we have previously demonstrated that this method is sufficiently sensitive for the quantification of basal NO in resistance and conduit arteries [19, 28, 29]. Continuous hypoxia induced a larger reduction of NO availability, compared to intermittent exposure. This can be explained by a reduced NO production, as suggested by the decreased expression of phosphorylated eNOS—the active form of the enzyme—found in chronic hypoxia only. The lack of effect of intermittent exposure could be explained by the functional “on-off” (hypoxia-normoxia) switch [30] which is not able to induce downregulation of the NO biosynthetic machinery.

Reduced NO availability in hypoxic conditions seems also to be related to NO destruction by O_2_^∙−^. This is suggested by the increased superoxide anion production found in CIH2x2 and CH pulmonary arteries, similarly to data described in an OSA rat model [31]. Superoxide anion levels were even higher in continuous compared to intermittent hypoxia, suggesting that continuous exposure further stimulates the enzymatic systems responsible for ROS synthesis. In the vascular wall, several enzymes produce O_2_^∙−^, NADPH oxidase being the main system in the pulmonary vasculature [13, 32]. We found a remarkable expression of this enzyme—confirmed by immunohistochemistry—in both CH and CIH2x2, while it was very low in normoxic rats. The important role of this enzyme in intermittent hypoxia has been previously demonstrated in NADPH-oxidase knockout mice exposed to OSA [33]. Our data show that O_2_^∙−^ levels were larger in continuous compared to intermittent hypoxia, despite the similar p22phox expression in both groups. This could be explained by O_2_^∙−^ production by xanthine oxidase, as suggested in OSA patients and animal models [34, 35]. Since it has been reported that, under hypoxic conditions, O_2_^∙−^ generated by xanthine oxidase is very small [36], alternatively, O_2_^∙−^ can be produced by “uncoupled” dysfunctional eNOS, which can be induced by peroxynitrite [37]. We did not measure peroxynitrite directly but quantified 3-nitrotyrosine, which is currently accepted as evidence of peroxynitrite generation. The larger content of 3-nitrotyrosine found in CH suggests that eNOS uncoupling can contribute to larger O_2_^∙−^ production found in continuous hypoxic conditions compared to intermittent conditions, despite the similar NADPH oxidase expression in both groups.

Oxidative-nitrosative stress is associated with vascular remodeling in hypoxia-induced pulmonary hypertension [38]. We have previously found several signs of remodeling in pulmonary arteries from CH and CIH2x2 rats, including wall hypertrophy due to increased smooth muscle and adventitial cells [15, 16]. Adventitial NADPH oxidase has been described to be involved in pulmonary artery adventitial fibroblasts proliferation [39, 40] and seems to be a primary site of superoxide anion production in the vessel wall [41–43]. We found that chronic or intermittent hypoxia substantially increased adventitial cell number together with a larger percentage of NADPH-positive cells. Moreover, we [44] and others [43] have previously demonstrated that adventitia is a key layer regarding NO inactivation by ROS. Since NO is an antiproliferative agent, we suggest that an imbalance between NO/O_2_^∙−^ can be linked to the vascular remodeling process under continuous or intermittent hypoxic exposure.

It was beyond the scope of this study to investigate the mechanisms implicated in hypoxia-induced NO/ROS disbalance. However, we can speculate on the possible role of inflammation. Alveolar hypoxia produces widespread systemic inflammation [45] and it also promotes the development of a pulmonary artery chronic inflammatory microenvironment [46]. We also have evidence of infiltrated macrophages in pulmonary arteries from CH and CIH2x2 (unpublished results), suggesting that local inflammation in the vascular wall might contribute to the NO/ROS disbalance. In support of this hypothesis, there is evidence that tumor necrosis factor-*α* can reduce eNOS expression and activity in pulmonary arteries [47]. Moreover, inflammation associated with macrophage infiltration can also contribute to ROS generation through stimulation of NADPH oxidase expression, as previously found in the carotid body under intermittent hypoxic conditions [48].

In conclusion, the present study suggests that hypobaric hypoxia under intermittent conditions reduces NO availability due to destruction by superoxide anion, without affecting NO synthesis, while continuous exposure is associated with both increased degradation and reduced NO production. The oxidative-nitrosative stress induced by long term intermittent hypoxia might participate in the observed cardiovascular structural alterations but represents a milder form of damage compared to continuous exposure. These data suggest that the alterations in oxidative status of humans chronically or intermittently exposed to high altitude need to be evaluated, in order to improve the associated cardiovascular alterations.

## References

[1] León-Velarde F, Maggiorini M, Reeves JT (2005). Consensus statement on chronic and subacute high altitude diseases. *High Altitude Medicine and Biology*.

[2] Dumitrascu R, Heitmann J, Seeger W, Weissmann N, Schulz R (2013). Obstructive sleep apnea, oxidative stress and cardiovascular disease: lessons from animal studies. *Oxidative Medicine and Cellular Longevity*.

[3] Richalet J-P, Donoso MV, Jiménez D (2002). Chilean miners commuting from sea level to 4500 m: a prospective study. *High Altitude Medicine and Biology*.

[4] Foster GE, Brugniaux JV, Pialoux V (2009). Cardiovascular and cerebrovascular responses to acute hypoxia following exposure to intermittent hypoxia in healthy humans. *Journal of Physiology*.

[5] Siqués Lee P, Brito J, León-Velarde F (2006). Time course of cardiovascular and hematological responses in rats exposed to chronic intermittent hypobaric hypoxia (4600 m). *High Altitude Medicine and Biology*.

[6] Corno AF, Milano G, Morel S (2004). Hypoxia: unique myocardial morphology?. *Journal of Thoracic and Cardiovascular Surgery*.

[7] McGuire M, Bradford A (1999). Chronic intermittent hypoxia increases haematocrit and causes right ventricular hypertrophy in the rat. *Respiration Physiology*.

[8] Brito J, Siqués P, León-Velarde F (2008). Varying exposure regimes to long term chronic intermittent hypoxia exert different outcomes and morphological effects on Wistar rats at 4600 m. *Toxicological and Environmental Chemistry*.

[9] Durmowicz AG, Stenmark KR (1999). Mechanisms of structural remodeling in chronic pulmonary hypertension. *Pediatrics in Review*.

[10] Sommer N, Dietrich A, Schermuly RT (2008). Regulation of hypoxic pulmonary vasoconstriction: basic mechanisms. *European Respiratory Journal*.

[11] Wolin MS, Gupte SA, Mingone CJ, Neo BH, Gao Q, Ahmad M (2010). Redox regulation of responses to hypoxia and NO-cGMP signaling in pulmonary vascular pathophysiology. *Annals of the New York Academy of Sciences*.

[12] Steudel W, Scherrer-Crosbie M, Bloch KD (1998). Sustained pulmonary hypertension and right ventricular hypertrophy after chronic hypoxia in mice with congenital deficiency of nitric oxide synthase. *Journal of Clinical Investigation*.

[13] Frazziano G, Champion HC, Pagano PJ (2012). NADPH oxidase-derived ROS and the regulation of pulmonary vessel tone. *The American Journal of Physiology*.

[14] Griendling KK, Ushio-Fukai M (2000). Reactive oxygen species as mediators of angiotensin II signaling. *Regulatory Peptides*.

[15] González MC Confocal Microscopy as New Tool for the Study of Pulmonary Artery Remodelling at a Cellular Level in Rats Exposed to Chronic Hypobaric Hypoxia.

[16] Siques P (2010). Structural changes in pulmonary artery of exposed rats to chronic intermittent hypobaric hypoxia. *High Altitude Medicine and Biology*.

[17] Germack R, Leon-Velarde F, Valdes De La Barra R, Farias J, Soto G, Richalet JP (2002). Effect of intermittent hypoxia on cardiovascular function, adrenoceptors and muscarinic receptors in Wistar rats. *Experimental Physiology*.

[18] Brito J, Siqués P, León-Velarde F, De La Cruz JJ, López V, Herruzo R (2007). Chronic intermittent hypoxia at high altitude exposure for over 12 years: assessment of hematological, cardiovascular, and renal effects. *High Altitude Medicine and Biology*.

[19] González JM, Somoza B, Conde MV, Fernández-Alfonso MS, González MC, Arribas SM (2008). Hypertension increases middle cerebral artery resting tone in spontaneously hypertensive rats: role of tonic vasoactive factor availability. *Clinical Science*.

[20] Wang Z, Li AY, Guo QH (2013). Effects of cyclic intermittent hypoxia on ET-1 responsiveness and endothelial dysfunction of pulmonary arteries in rats. *PLoS ONE*.

[21] Tahawi Z, Orolinova N, Joshua IG, Bader M, Fletcher EC (2001). Altered vascular reactivity in arterioles of chronic intermittent hypoxic rats. *Journal of Applied Physiology*.

[22] Phillips SA, Olson EB, Morgan BJ, Lombard JH (2004). Chronic intermittent hypoxia impairs endothelium-dependent dilation in rat cerebral and skeletal muscle resistance arteries. *The American Journal of Physiology*.

[23] Irwin DC, McCord JM, Nozik-Grayck E (2009). A potential role for reactive oxygen species and the HIF-1*α*-VEGF pathway in hypoxia-induced pulmonary vascular leak. *Free Radical Biology and Medicine*.

[24] Weissmann N, Schermuly RT, Ghofrani HA (2006). Hypoxic pulmonary vasoconstriction: triggered by an increase in reactive oxygen species?. *Novartis Foundation Symposium*.

[25] Weissmann N, Zeller S, Schäfer RU (2006). Impact of mitochondria and NADPH oxidases on acute and sustained hypoxic pulmonary vasoconstriction. *The American Journal of Respiratory Cell and Molecular Biology*.

[26] Kojima H, Nakatsubo N, Kikuchi K (1998). Direct evidence of NO production in rat hippocampus and cortex using a new fluorescent indicator: DAF-2 DA. *NeuroReport*.

[27] Yi F-X, Zhang AY, Campbell WB, Zou A-P, Van Breemen C, Li P-L (2002). Simultaneous in situ monitoring of intracellular Ca2+ and NO in endothelium of coronary arteries. *The American Journal of Physiology*.

[28] Arribas SM, Daly CJ, González MC, Mcgrath JC (2007). Imaging the vascular wall using confocal microscopy. *Journal of Physiology*.

[29] Somoza B, Abderrahim F, González JM (2006). Short-term treatment of spontaneously hypertensive rats with liver growth factor reduces carotid artery fibrosis, improves vascular function, and lowers blood pressure. *Cardiovascular Research*.

[30] Powell FL, Garcia N (2000). Physiological effects of intermittent hypoxia. *High Altitude Medicine and Biology*.

[31] Norton CE, Jernigan NL, Kanagy NL, Walker BR, Resta TC (2011). Intermittent hypoxia augments pulmonary vascular smooth muscle reactivity to NO: regulation by reactive oxygen species. *Journal of Applied Physiology*.

[32] Fuchs B, Sommer N, Dietrich A (2010). Redox signaling and reactive oxygen species in hypoxic pulmonary vasoconstriction. *Respiratory Physiology and Neurobiology*.

[33] Nisbet RE, Graves AS, Kleinhenz DJ (2009). The role of NADPH oxidase in chronic intermittent hypoxia-induced pulmonary hypertension in mice. *The American Journal of Respiratory Cell and Molecular Biology*.

[34] Dopp JM, Philippi NR, Marcus NJ (2011). Xanthine oxidase inhibition attenuates endothelial dysfunction caused by chronic intermittent hypoxia in rats. *Respiration*.

[35] El Solh AA, Saliba R, Bosinski T, Grant BJB, Berbary E, Miller N (2006). Allopurinol improves endothelial function in sleep apnoea: a randomised controlled study. *European Respiratory Journal*.

[36] Al Ghouleh I, Khoo NKH, Knaus UG (2011). Oxidases and peroxidases in cardiovascular and lung disease: new concepts in reactive oxygen species signaling. *Free Radical Biology and Medicine*.

[37] Förstermann U (2010). Nitric oxide and oxidative stress in vascular disease. *Pflügers Archiv*.

[38] Aggarwal S, Gross CM, Sharma S, Fineman JR, Black SM Reactive oxygen species in pulmonary vascular remodeling. *Comprehensive Physiology*.

[39] Panzhinskiy E, Zawada WM, Stenmark KR, Das M (2012). Hypoxia induces unique proliferative response in adventitial fibroblasts by activating PDGFbeta receptor-JNK1 signalling. *Cardiovascular Research*.

[40] Li S, Tabar SS, Malec V (2008). NOX4 regulates ROS levels under normoxic and hypoxic conditions, triggers proliferation, and inhibits apoptosis in pulmonary artery adventitial fibroblasts. *Antioxidants and Redox Signaling*.

[41] Berry C, Hamilton CA, Brosnan MJ (2000). Investigation into the sources of superoxide in human blood vessels: angiotensin II increases superoxide production in human internal mammary arteries. *Circulation*.

[42] Pagano PJ, Ito Y, Tornheim K, Gallop PM, Tauber AI, Cohen RA (1995). An NADPH oxidase superoxide-generating system in the rabbit aorta. *The American Journal of Physiology*.

[43] Wang HD, Pagano PJ, Du Y (1998). Superoxide anion from the adventitia of the rat thoracic aorta inactivates nitric oxide. *Circulation Research*.

[44] Somoza B, González MC, González JM, Abderrahim F, Arribas SM, Fernández-Alfonso MS (2005). Modulatory role of the adventitia on noradrenaline and angiotensin II responses: role of endothelium and AT2 receptors. *Cardiovascular Research*.

[45] Chao J, Wood JG, Blanco VG, Gonzalez NC (2009). The systemic inflammation of alveolar hypoxia is initiated by alveolar macrophage-borne mediator(s). *The American Journal of Respiratory Cell and Molecular Biology*.

[46] Burke DL, Frid MG, Kunrath CL (2009). Sustained hypoxia promotes the development of a pulmonary artery-specific chronic inflammatory microenvironment. *The American Journal of Physiology*.

[47] Zhang J, Patel JM, Li YD, Block ER (1997). Proinflammatory cytokines downregulate gene expression and activity of constitutive nitric oxide synthase in porcine pulmonary artery endothelial cells. *Research Communications in Molecular Pathology and Pharmacology*.

[48] Lam S-Y, Liu Y, Ng K-M (2012). Chronic intermittent hypoxia induces local inflammation of the rat carotid body via functional upregulation of proinflammatory cytokine pathways. *Histochemistry and Cell Biology*.

